# Adipsin of the Alternative Complement Pathway Is a Potential Predictor for Preeclampsia in Early Pregnancy

**DOI:** 10.3389/fimmu.2021.702385

**Published:** 2021-10-04

**Authors:** Min Liu, Xiaolei Luo, Qin Xu, Hongbiao Yu, Linbo Gao, Rong Zhou, Tao Wang

**Affiliations:** ^1^ Department of Obstetrics and Gynecology, Center for Translational Medicine, Key Laboratory of Birth Defects and Related Diseases of Women and Children (Sichuan University), Ministry of Education, West China Second University Hospital, Sichuan University, Chengdu, China; ^2^ Center for Translational Medicine, Key Laboratory of Birth Defects and Related Diseases of Women and Children (Sichuan University), Ministry of Education, Department of Obstetrics and Gynecology, West China Second University Hospital, Sichuan University, Chengdu, China

**Keywords:** preeclampsia, complement system, biomarker, adipsin, sENG

## Abstract

**Objective:**

The concentrations of complement proteins (adipsin, C3a, and C5a) and soluble endoglin (sENG) in the plasma were measured in this study, and their value as early-pregnancy predictors and potential diagnostic marker of preeclampsia was assessed, respectively.

**Experimental Design:**

Plasma samples were obtained from healthy and preeclampsia pregnant women before delivery for a cross-sectional study. Plasma samples were collected from healthy and preeclampsia pregnant women throughout pregnancy and postpartum for a follow-up study. Enzyme-linked immunosorbent assays were used to detect plasma levels of several complement proteins (adipsin, C3a, and C5a) and sENG.

**Results:**

The plasma levels of adipsin, C5a, and sENG were significantly increased before delivery in pregnant women with preeclampsia. During pregnancy, the plasma adipsin, C5a, and sENG levels were increased from the third trimester in healthy pregnant women; plasma adipsin levels remained stable after delivery, while C3a levels increased in the second trimester and remained stable afterward. Furthermore, levels of adipsin, C5a, and sENG were higher in preeclampsia patients at different stages of pregnancy; the C3a level presents a similar change and no difference was found in the third trimester. In the first trimester, receiver-operating curve (ROC) curve analysis showed that adipsin (AUC, 0.83 ± 0.06, *P*=0.001) and sENG (AUC, 0.74 ± 0.09, *P*=0.021) presented high value as predictors of early pregnancy.

**Conclusions:**

Adipsin is likely a novel plasma biomarker to monitor the increased risk of preeclampsia in early pregnancy. Moreover, the increased plasma levels of adipsin, C5a, and sENG before delivery may be associated with preeclampsia.

## Introduction

Preeclampsia is a pregnancy complication mainly characterized by gestational hypertension, proteinuria, systemic endothelial cell activation, and inflammatory overreaction (1). Preeclampsia affects 3–5% of pregnancies, is higher in low-resource settings (2), and contributes significantly to maternal and neonatal mortality and morbidity (3). Although the etiology of preeclampsia remains poorly understood, it is believed that the immune system is involved in its pathogenesis, which needs further clarification (4).

Recent studies have found that dysregulation of the complement system contributed to the pathogenesis of preeclampsia (reviewed in (5)). The complement system has functions critical to the innate immune response which is activated when the embryo attaches *in utero* because of the heterogenicity of an embryo relative to its mother (6). The complement cascade has three initiating mechanisms, including the classical, lectin, and alternative pathways (7). Adipsin, also known as complement Factor D, is expressed and secreted at high levels by adipose tissue and is a key molecule in the alternative pathway (8). Natalia et al. reported that levels of adipsin were significantly elevated in pregnant women with preeclampsia in the last trimester (9). Our previous study also found that the adipsin levels were significantly elevated in the plasma of patients with preeclampsia, and the urinary adipsin concentration appeared to be a good biomarker for the diagnosis of preeclampsia (10). We hypothesized that changes in plasma adipsin levels might be valuable for prediction or diagnosis.

The main function of adipsin is to catalyse the breakdown of complement factor C3 (11). These suggest that adipsin may affect the downstream molecules such as C3a and C5a by participating in alternative pathway activation. In alternative pathways, free C3a and C5a are formed from the C3 and C5 complements and released into the circulation, companying with complement activation (12). Richani et al. showed that the concentrations of C3a, C4a, and C5a in maternal plasma were higher in normal pregnant women than in non-pregnant women, and the concentration of C3a, C4a, and C5a did not change with gestational age (13), it indicates complement system is activated during the normal pregnancy. Haeger et al. reported that plasma C3a and C5a concentrations in patients with preeclampsia were higher than in normal pregnant women at the time of delivery (14, 15). However, other studies showed that the C3a level had no significant difference between healthy and preeclampsia pregnant women (16, 17). Hence, previous studies did not achieve consistent conclusions about the plasma level of C3a in preeclamptic patients compared to pregnant controls.

Biomarkers in maternal blood seem to have a modest predictive potential in early pregnancy or have good prediction for preeclampsia (18). Accumulating evidence suggests that preeclampsia results from an imbalance in angiogenic factors, which damage maternal vascular endothelium (19, 20). It is known that circulating concentrations of soluble endoglin (sENG) seem to be a suitable marker to assess the severity of preeclampsia (21). However, published reports regarding the levels of sENG at different gestation stages are scarce.

In this study, the levels of adipsin, C3a, C5a, and sENG before delivery were measured to assess their role in preeclampsia. Then, a follow-up analysis was conducted to determine whether complement levels and sENG fluctuate with gestational age and whether plasma adipsin and related important circulating complement molecules can be used as an early-pregnancy predictor and potential diagnostic biomarkers of preeclampsia.

## Materials and Methods

The research protocol was approved by the Institutional Committee for the Protection of Human Subjects (the Institutional Review Board of West China Second University Hospital, Sichuan University), and all included patients received routine prenatal examinations and provided informed consent. A cross-sectional study was conducted with 33 patients with severe preeclampsia and 32 controls with a healthy pregnancy without hypertension from a cohort of pregnant women before delivery. Then, a follow-up study subjects were enrolled pregnant during different stages of pregnancy from September 2018 to March 2020, as described in the following. Healthy subjects: first trimester (n=35) (90.20 ± 4.99 days of gestation, range: 80–101); second trimester (n=31) (173.51 ± 8.05 days of gestation, range: 164–196); third trimester (n=35) (229.37 ± 12.84 days of gestation, range: 212–272); postpartum period (n=32) (3 days after delivery); and preeclampsia subjects: first trimester (n=11) (93.64 ± 8.23 days of gestation, range: 78–113); second trimester (n=10) (182.55 ± 10.53 days of gestation, range: 172–204); third trimester (n=10) (225.73 ± 10.75 days of gestation, range: 212–257); postpartum period (n=10) (3 days after delivery). **Table 1** presents the clinical characteristics of the enrolled pregnant women in this cross-sectional study and the follow-up study.

**Table 1:** Clinical characteristics of cross-sectional study subjects.

Characteristics	Cross-sectional study		Follow-up study	
	Healthy pregnant women (n = 32)	Preeclamptic patients (n = 33)	Healthy pregnant women (n = 35)	Preeclamptic patients (n = 11)
Maternal age (years)	32.66 ± 3.68 (26–43)	30.00 ± 4.23 (22–39)*	31.54 ± 3.25 (23–37)	31.36 ± 3.96 (24–39)
Pre-pregnancy BMI (kg/m^2^)	25.98 ± 2.31 (21.08–29.40)	27.64 ± 3.54 (21.93–33.06)*	25.36 ± 2.74 (19.77–30.49)	28.88 ± 3.22 (23.83–32.23)*
Primagravida	20	23	20	8
Current smoking	0	0	0	0
Diabetes mellitus	1	1	1	0
Gestational age at delivery (days)	272.94 ± 7.34 (258–289)	227.48 ± 27.06 (185–262)**	272.63 ± 13.32 (216–291)	263.82 ± 21.19 (202–285)
Systolic blood pressure (mm Hg)	113.78 ± 12.48 (94–123)	152.67 ± 11.10 (142–188)*	119.20 ± 9.36 (97–130)	149.73 ± 9.04 (139–168)**
Diastolic blood pressure (mm Hg)	73.53 ± 8.74 (54–88)	97.67 ± 7.92 (81–117)**	72.60 ± 7.89 (55–86)	99.45 ± 7.08 (94–115)**
24-h urinary protein (g)	**/**	2.93 ± 2.81 (0.24–10.60)**	**/**	1.31 ± 1.78 (0.24–6.34)**

BMI indicates body mass index.

Comparisons between groups were performed using Student’s t-test, and results are presented as mean ± SD. *P < 0.05, **P < 0.01, healthy vs. preeclamptic pregnant women.

The diagnosis of preeclampsia is described in a previous study (22). Briefly, preeclampsia is defined new onset of hypertension present after 20 weeks of gestation combined with systolic blood pressure (BP) ≥140 mm Hg or diastolic BP ≥90 mm Hg on two occasions at least 4 hours apart while the patient is on bed rest, or proteinuria of ≥0.3 g in a 24-h urine specimen. Control subjects had blood pressure measurements taken on the day of enrollment and additional measurements at subsequent antenatal visits to ensure proper group assignment. Exclusion criteria including multiple pregnancies and transplanted organs, other pregnancy complications except for diabetes mellitus (e.g., chronic hypertension), other complications (e.g., renal diseases, oncological diseases and autoimmune diseases), and any known fetal anomalies, were excluded.

### Sample Collection

PASS Sample Size Software was used to estimate sample size, two-sample *t*-test power analysis was performed according to the results of preliminary experiment. The blood was collected in a sterile EDTA-containing vacutainer tube, and serial blood samples were collected from pregnant women enrolled in the follow-up study. The blood samples were kept at room temperature for 20 min, and then samples were centrifuged at 500g and 4°C for 10 min. The plasma was collected and stored at -80°C until further analysis.

### Enzyme-Linked Immunosorbent Assay

sENG, adipsin, C3a, and C5a in plasma samples were determined in duplicate by ELISA using commercial kits purchased from RayBiotec, Inc (Norcross, GA). The experiments were conducted in accordance with the manufacturer’s protocols as described previously (23).

### Statistical Analysis

Data analysis was performed using SPSS 19 (SPSS Science Inc., Chicago, Illinois) and Prism 6.0 (Graph Pad Software, La Jolla, CA, USA). The unpaired *t*-test was used to test for normal distribution of the data. Since the maternal plasma concentrations of C5a were not normally distributed, nonparametric Mann–Whitney *t*-tests were used for analyses. The levels of sENG, adipsin, and C3a in plasma were expressed as mean ± SD values. *P*<0.05 was considered significant. Binary logistic regression was used to examine the association between plasma adipsin levels and the risk of developing preeclampsia by calculating unadjusted and adjusted odds ratios (ORs). The diagnostic value of these complements for preeclampsia was determined by using receiver-operating characteristics (ROC) curves.

## Results

### Levels of sENG, adipsin, C3a, and C5a in the Plasma of Pregnant Women With Preeclampsia and Healthy Pregnant Women Before Delivery

**Figure 1:**
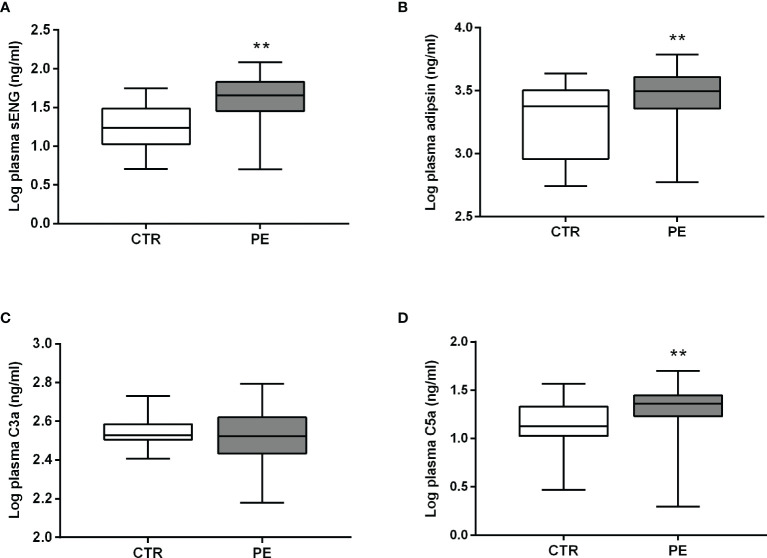
Plasma levels of sENG, adipsin, C3a, and C5a before delivery. Levels of sENG **(A)**, adipsin **(B)**, C3a **(C)**, and C5a **(D)** in the plasma of healthy (white, n = 32) and preeclampsia (gray, n = 33) populations as measured using ELISA. ^**^ *P* < 0.01 *vs*. healthy pregnant women.

Levels of sENG, adipsin, and C5a in the plasma of pregnant women with preeclampsia were higher than those in healthy pregnant women before delivery (**Figures 1A, B, D**). The level of sENG in pregnant women with preeclampsia (48.44 ± 5.018 ng/mL) was significantly higher than that in healthy pregnant women (21.59 ± 2.358 ng/mL) (*P*<0.001). The adipsin concentrations were 2153 ± 201.4 ng/mL and 3161 ± 214.7 ng/mL in the healthy and preeclampsia populations, respectively (*P*<0.01). However, there was no significant change in the C3a levels amongst patients with preeclampsia (**Figure 1C**). In addition, to exclude the effect of BMI on changes of adipsin levels, further statistical analysis was performed. After adjusting this difference for BMI, plasma level of adipsin remained significantly lower in the preeclampsia group (**Table 2**, *P<*0.05).

**Table 2:** Early predictive value of sENG, adispin, C5a, and C3a for preeclampsia.

	sENG	adipsin	C3a	C5a	adipsin + sENG
Sensitivity (Se) (%)	72.7	81.8	63.6	72.7	90.9
Specificity (Sp) (%)	60.6	75.8	66.7	57.6	48.0
Positive predictive value (PPV) (%)	38.1	52.9	38.9	36.4	47.1
Negative predictive value (NPV) (%)	86.4	92.3	84.0	85.7	88.9

### Levels of sENG, adipsin, C3a, and C5a in the Plasma During Healthy and Preeclampsia Pregnancy

**Figure 2:**
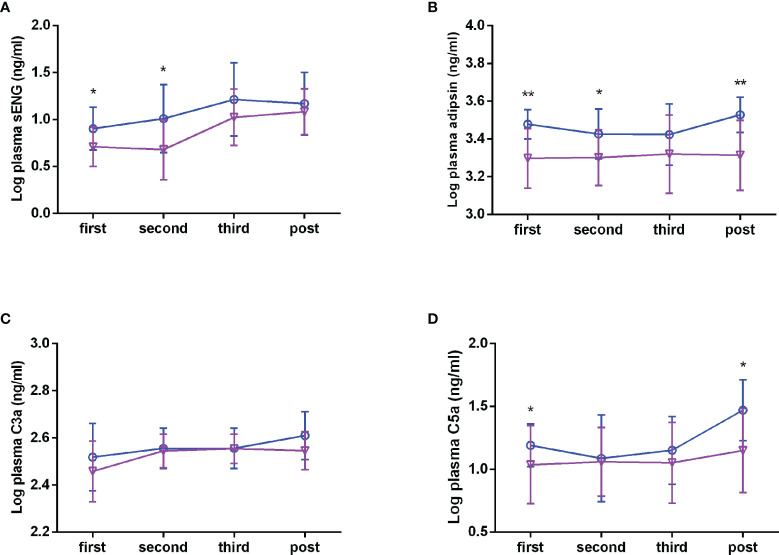
Plasma levels of sENG, adipsin, C3a, and C5a during the pregnancy. sENG **(A)**, adipsin **(B)**, C3a **(C)**, and C5a **(D)** in the plasma samples of pregnant women with preeclampsia and healthy pregnant women throughout pregnancy were tested using ELISA kits, including in the first trimester (healthy n = 35, preeclampsia n = 11); second trimester (healthy n = 31, preeclampsia n=10); third trimester (healthy n=35, preeclampsia n = 10); and the postpartum period (healthy n = 32, preeclampsia n = 10). **σ** full line: healthy, **○** dotted line: preeclampsia. ^*^ *P* < 0.05, ^**^ *P* < 0.01 *vs*. healthy pregnant women.

To explore whether circulating complements and sENG levels fluctuate with gestational age in healthy subjects, we detected the concentrations during the different stages of pregnancy. As shown in **Figures 2A–C**, in healthy subjects, the plasma sENG level was increased from the third trimester, the plasma adipsin level had no significant difference throughout the pregnancy, and the levels of C3a was increased in the second trimester and remained stable thereafter. Next, we compared the differences of circulating complements and sENG levels between healthy and preeclampsia subjects at different gestation stages. Compared with the healthy subjects, plasma sENG levels showed an increase in pregnant women with preeclampsia throughout the pregnancy, but there were significant differences only in first and second trimesters (**Figure 2A**, *P<*0.05). Levels of adipsin were higher in pregnant women with preeclampsia throughout the pregnancy, but had no significant difference in third trimester (**Figure 2B**, *P<*0.05, *P<*0.01). C3a levels showed rising trends (**Figure 2C**); C5a levels were not significantly different throughout normal pregnancy, but rather increased significantly in first trimester and after delivery in women with preeclampsia (**Figure 2D**, P<0.05).

### Predictive Value of sENG, adipsin, C3a, and C5a in Early Pregnancy for Preeclampsia

**Figure 3:**
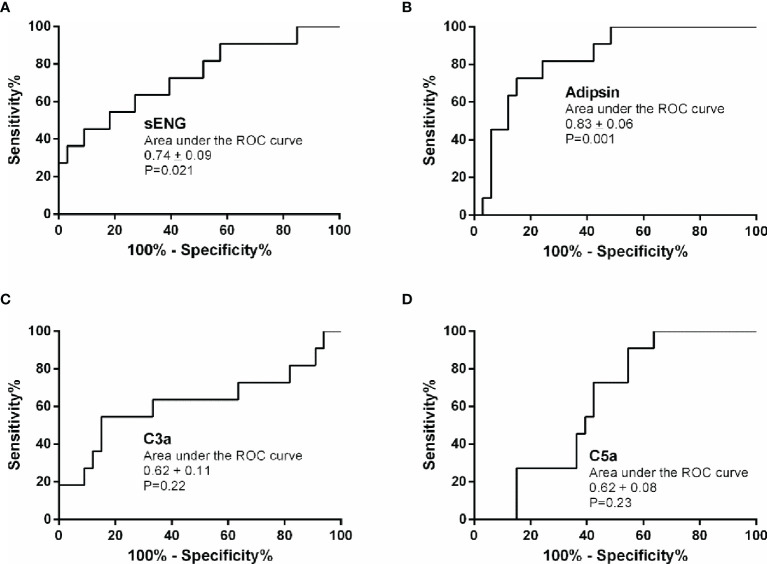
Predictive value of sENG, adipsin, C3a, and C5a for preeclampsia. Receiver-operating characteristics (ROC) curves were plotted for sENG **(A)**, adipsin **(B)**, C3a **(C)**, and C5a **(D)**.

Based on the plasma levels of the complement molecules and sENG from the healthy control and preeclampsia cases, their predictive values were evaluated using the area under the ROC curve (AUC). The results are presented in **Table 2** and **Figure 3**. Compared with the reference factor sENG (AUC, 0.74 ± 0.09; **Figure 3A**, *P*=0.021), the predictive value of adipsin (AUC, 0.83 ± 0.06; **Figure 3B**, *P*=0.001) showed better. Eleven patients with preeclampsia were followed up, and their onset time was 37.09 ± 2.82 weeks of gestation. In early pregnancy, plasma adipsin levels of 10 patients were higher than the average level of healthy subjects (2110.97 ± 740.09 ng/mL), and nine of them were diagnosed with preeclampsia in the third trimester, proving that adipsin has a good predictive value.

The threshold that provided maximal sensitivity and specificity for the prediction of preeclampsia was determined as the cut-off value. As shown in **Table 3**, the sensitivity and specificity of adipsin was the highest, at 81.8% and 75.8%, followed by that of sENG, at 72.7% and 60.6%, respectively. Moreover, the positive predict value and negative predict value of adipsin also was the highest, at 52.9% and 92.3%, respectively. Because adipsin showed a much higher sensitivity and specificity, we decided to use adipsin as a basis for combinations. The results showed that the sensitivity of adipsin and sENG was the highest at 90.9%, but the specificity decreased to 48.0%.

**Table 3:** The unadjusted and adjusted ORs with 95% CIs of the relationship between preeclampsia and BMI.

Groups	n	Unadjusted OR (95% CI)	*P* value	Adjusted OR (95% CI)	*P* value
Control	32	1	<0.05	1	<0.05
Preeclampsia	33	3.28 (1.11-9.71)		5.16 (1.45-18.33)	

The BMI (control vs preeclampsia, 25.98 ± 2.31 vs 27.64 ± 3.54, P<0.05) was not regarded as a potential confounder in the analysis of adipsin levels between preeclampsia and control groups. BMI, body mass index; ORs, odd ratios; CIs, confidence intervals.

## Discussion

Accumulating data from the clinical research support that biomarkers contribute with diagnostically relevant information, also in the early disease stages (24). Angiogenic and anti-angiogenic factors have emerged as important biomarkers in preeclampsia. This seminal discovery has led to many biomarker studies, attempting to predict preeclampsia with PlGF, sFlt-1, and the sFlt-1/PlGF ratio, as well as to predict the absence of preeclampsia in pregnant women (25). In women with suspected preeclampsia presenting at <34 weeks, circulating sFlt1/PlGF ratio predicts adverse outcomes occurring within 2 weeks (26). It is biologically plausible that sENG is a blood-based biomarker of placental dysfunction (27). Moreover, changes in the levels of sENG and sFlt-1 between the first and second trimesters were predictive of preterm preeclampsia (28). Circulating sENG levels increased markedly beginning 2–3 months before the onset of preeclampsia (29). However, their position is still inconclusive in the early prediction and diagnosis of disease. Considering that sFlt-1 is generally detected simultaneously with PlGF, and the ratio of sFlt-1/PlGF needs to be calculated. This is a little bit more complicated. Moreover, the constitutive levels of plasma sFlt-1 and PlGF are lower, and more samples are needed. Many studies have shown that sENG also as a good biomarker for preeclampsia (21, 27), and the constitutive level of sENG is higher and stable. In this study, we also found that plasma sENG levels were significantly higher in patients with preeclampsia than in those with healthy pregnancy. Unlike in the past, we found that the plasma sENG levels of preeclampsia subjects were increased throughout the pregnancy, and returned to their normal levels after delivery. This further confirms the importance of sENG for early-pregnancy prediction and diagnosis in preeclampsia.

A series of studies have shown that changes in the serum levels of complement proteins (C1q, Bb, and C5b-9) could be potential diagnostic markers for severe preeclampsia (30, 31). Lynch et al. conducted a prospective study in human pregnancy (n=701) to investigate whether elevated levels of complement activation fragment Bb (reflecting activation of alternative complement pathway) at a single point in early pregnancy (>20 weeks gestation) were predictive of preeclampsia later in pregnancy (31). There was also a strong correlation between urinary levels of membrane attack complex C5b-9 and urinary excretion of sFlt-1. Urinary C5b-9 is a promising biomarker in severe preeclampsia (32). However, the changes of sC5b-9 in plasma were not significant. It reported that the level of sC5b-9 did not increase significantly in the plasma of patients with preeclampsia throughout pregnancy (33). Agostinis et al. also indicated that the plasma level of the sC5b-9 did not change significantly between the preeclampsia and healthy pregnant women (34). Thus, the sC5b-9 may not be a possible predictor in plasma of early pregnancy, it was not measured in this study. This suggested that complement component fragments have the potential and application value as a biomarker. Our previous study also found that the adipsin levels were significantly elevated in the plasma of patients with preeclampsia, and the urinary adipsin concentration seems to be a good biomarker for the diagnosis of preeclampsia (10). Indeed, we found adipsin and C5a levels in plasma were increased before delivery, similar to the sENG level. Therefore, we speculated that changes in plasma complement levels might be valuable for prediction in early pregnancy.

Components of the alternative pathway are important for successful placentation, and appropriate regulation of complement activation is critical for pregnancy (7). Activation of the alternative pathway might play a connecting role between placentally derived inflammatory stimuli and the maternal syndrome of preeclampsia (35–37). Although studies have shown that the complement-activation fragments in maternal plasma such as Bb, C3a, C5a are a significant risk factor for preeclampsia (14, 16), only few studies focused on the upstream complement protein such as adipsin. Adipsin is related to the activation of the complement pathway in the decidual fibroblast, which contributes to the activation of the defense mechanisms of the extracellular placental tissue in mouse (38). Recently, it has also been indicated that the adipsin-C3a pathway connects adipocyte function to β-cell physiology (39). We found that levels of adipsin are higher in pregnant women with preeclampsia throughout the pregnancy. The increased level of adipsin towards late gestation may be involved in pregnancy-associated metabolic changes and the pathophysiology of preeclampsia (9). Therefore, the increase in adipsin levels may regulate changes in downstream products and participate in vascular endothelial injury in preeclampsia patients. Given that the plasma adipsin levels were increased from the first trimester, adipsin may play a role in the onset of preeclampsia and could therefore be used as a potential marker. Indeed, the sensitivity and specificity of adipsin was the highest, at 81.80% and 75.8%. This study followed up with 11 preeclampsia patients, and their onset time was 37.09 ± 2.82 weeks of gestation. In early pregnancy, the plasma adipsin levels of 10 patients were higher than the average plasma levels of healthy subjects, and nine of them were diagnosed with preeclampsia in the third trimester. Moreover, the combination of sENG and adipsin seems to be a more accurate biomarker to predict patients at risk for preeclampsia in early pregnancy, as the sensitivity of adipsin was 90.9%. Although the specificity of the combination was significantly reduced, it is still of great value for early prediction, as it can be combined with other clinical features as an auxiliary predictor. This is a clinically relevant finding but requires further validation in a research with large sample size.

It should note is that there is a significant difference in BMI between preeclampsia and control groups. Also, it was revealed that the circulating adipsin level tends to correlate positively with the BMI of individuals (40). A positive correlation with weight and BMI in the first and last period were found when adipsin levels were analyzed in each period of gestation (9). Reynolds et al. found significant associations between BMI≥25 and increased levels of fragments C3a and iC3b, and component CFH in the control subjects; however, there was no significant correlation between BMI and adipsin (41). We also found that the BMI was not regarded as a potential confounder in the analysis of adipsin levels between preeclampsia and control groups. Still, higher BMI is a known risk factor for the development of preelampsia and considered consistently to be a predictor of preeclampsia (42, 43). A large retrospective cohort study (18 years-survey, 1736 cases) indicated that the increment of BMI was only associated with late onset preeclampsia (44). Hence, adipsin can be used as a predictor of preeclampsia and is not affected by BMI, BMI is also a generally accepted predictor.

Many previous studies have reported the association between C3a level and preeclampsia with conflicting results. Research studies have indicated a lack of significant change in plasma C3a between normotensive and preeclamptic pregnancies (33, 45); these are in agreement with our present study findings. However, it is in contrast to other recent studies that showed elevated C3a in preeclampsia (35, 46, 47). Exogenous activation is particularly evident in blood with higher levels of extrinsic complement proteases (such as thrombin and tissue factor) that cleaved C3 and C5 *ex vivo* (48). Thus, the samples may have occurred complement activation *ex vivo*, leading to an inappropriate and misleading increases in C3a in the preeclamptic samples. Moreover, the differences in these results were related to the variety of complement components measured and the different detection methods employed.

A recent study suggested that targeting complement C5a promotes vascular integrity and limits airway remodeling, which is a key mechanism underlying the pathogenesis of severe preeclampsia (49). Our results showing that the C5a level was significantly increased in preeclampsia is in accordance with previous studies that demonstrated elevated plasma C5a levels just prior to delivery (45) and elevated maternal circulating C5a in preeclampsia throughout the gestational period (17). C5a and the terminal complex can activate monocytes and neutrophils with the release of biologically active and potent inflammatory mediators such as proteases, free oxygen radicals, and pro-inflammatory cytokines (50), which hinder angiogenesis and then contribute to placental insufficiency and maternal endothelial dysfunction in patients with preeclampsia (5).

In the current study, we analyzed the plasma levels of major complement components in women with normal pregnancy during the different stages of pregnancy. We found the plasma adipsin level remained stable throughout the pregnancy. This was in disagreement with the study that showed that a significant decrease in serum adipsin levels was observed throughout normal pregnancy compared with three months postpartum (9). We hypothesized that this may vary depending on the type and timing of the sample collected. Our finding is in accordance with previous studies which reported that there was a higher level of C3a in the second trimester, which then remained stable, while C5a levels remained largely unchanged (51). However, Derzsy et al. found that normal human pregnancy is characterized by a significant increase in C3a and C5a in the maternal circulation, which does not fluctuate with gestational age (15). It has been proposed that the inhibition of complement system promotes the physiologic changes at fetal-maternal interface required for a successful pregnancy, the complement levels of normal pregnancy are decreased in the early pregnancy compared to non-pregnant women. Then, some complements levels have increased trends or remain relatively stable throughout the pregnancy (52, 53). The results of follow-up study are similar with these findings. Instead, compared with the healthy pregnant women, the complements molecular levels of the preeclamptic pregnant women with smaller gestational age in the third trimester were higher. Thus, gestational age does not affect our result, that is, the complements values of preeclamptic pregnant women significantly were increased in third trimester.

In conclusion, we innovatively compared plasma levels of adipsin between the preeclampsia and control groups at different stages of pregnancy, and evaluated their predictive values in early pregnancy. The combination of sENG and adipsin seems to be a novel plasma biomarker to monitor the preeclampsia risk combined with known clinical predictors in early pregnancy. Moreover, the dysregulation of the complement system through the alternative pathway in the third trimester may be associated with preeclampsia. However, whether the change of complement molecular level is the direct cause of preeclamptic pathophysiology and the specific mechanism still need to be further investigated.

## Data Availability Statement

The raw data supporting the conclusions of this article will be made available by the authors, without undue reservation.

## Ethics Statement

The studies involving human participants were reviewed and approved by the Institutional Committee for the Protection of Human Subjects (the Institutional Review Board of West China Second University Hospital, Sichuan University, permit number 2019030). The patients/participants provided their written informed consent to participate in this study.

## Author Contributions

Conception and design: TW and RZ. Administrative support: TW. Provision of study materials or patients: QX and HY. Collection and assembly of data: ML and XL. Data analysis and interpretation: ML, XL, and LG. All authors contributed to the article and approved the submitted version.

## Funding

This study was financially supported by grants from Sichuan Science and Technology Program (No. 2018JY0551), and National Natural Science Foundation of China (No. 81871188, No. 81571465, No. 81871175).

## Conflict of Interest

The authors declare that the research was conducted in the absence of any commercial or financial relationships that could be construed as a potential conflict of interest.

## Publisher’s Note

All claims expressed in this article are solely those of the authors and do not necessarily represent those of their affiliated organizations, or those of the publisher, the editors and the reviewers. Any product that may be evaluated in this article, or claim that may be made by its manufacturer, is not guaranteed or endorsed by the publisher.
